# Evolving liver transplantation in Pakistan: Future challenges

**DOI:** 10.1016/j.amsu.2022.104669

**Published:** 2022-09-17

**Authors:** Abdul Wahab Dogar, Kaleem Ullah, Sidhant Ochani, Hafiz Bilal Ahmad

**Affiliations:** a Department of Liver Transplatation, Pir Abdul Qadir Shah Institute of Medical Sciences, Gambat, Pakistan; b Department of Medicine, Khairpur Medical College, Khairpur Mir's, Pakistan

## Abstract

Liver transplantation is a life-saving procedure that started in the early 60s. Initially, it struggled with multiple failed attempts but later it progressed and emerged as a gold standard procedure for liver failure secondary to various etiologies. In the first two decades, it faced various challenges like peri and post-operative care management, the quality of graft, optimal immunosuppressant use, and recipient selection criteria. Because of these challenges, the 1-year survival remained below 30% initially. Liver transplantation flourished tremendously over time due to advancements in organ preservation techniques, immunosuppressive therapies, and a better understanding of immunology. The invention of calcineurin inhibitors changed the dynamics of immunosuppressive therapies altogether. The donor organ shortage remains the leading challenge, Deceased donor transplantation activities have not yet started here due to various socio-cultural, and religious resistance. The majority population of the country is underprivileged and faces financial issues to afford the costly liver transplant procedure. The other challenges include the emerging NASH and obesity epidemics. The prevalence of viral hepatitis has not decreased in the country despite advancements in antiviral therapies and vaccine availability against hepatitis B. The local transplant community needs to overcome the limitation of organ supply through various donor expansion approaches. Although it may seem difficult to address all these challenges, still, the transplant community and health authorities need to find an effective way to sort out all these challenges.

Respected editor,

Liver transplantation is a life-saving procedure that started in the early 60s. Initially, it struggled with multiple failed attempts but later it progressed and emerged as a gold standard procedure for liver failure secondary to various etiologies [1]. In the first two decades, it faced various challenges like peri and post-operative care management, the quality of graft, optimal immunosuppressant use, and recipient selection criteria. Because of these challenges, the 1-year survival remained below 30% initially [2].

Liver transplantation flourished tremendously over time due to advancements in organ preservation techniques, immunosuppressive therapies, and a better understanding of immunology. The invention of calcineurin inhibitors changed the dynamics of immunosuppressive therapies altogether [3]. Similarly, the introduction of the brain death concept provided a more controlled environment of procurement which ultimately improved graft quality [4]. The evolution in the recipient selection criteria and advances in surgical techniques further enhanced the recipient's survival rate. All this led to the expansion of liver transplantation from only a few centers to more than a hundred programs in more than 80 countries of the world. Also, the graft outcomes continued to improve and the 1-year survival rate exceeded 80% [5].

In Pakistan, liver transplantation started a decade ago and it rapidly progressed over the last few years. Multiple centers have been established in the country and are increasing at a good pace. All these centers are actively performing living donor liver transplantation. Despite the early success of liver transplantation in the country, various challenges do persist.

The donor organ shortage remains the leading challenge in the country [6]. Deceased donor transplantation activities have not yet started here due to various socio-cultural, and religious resistance. The majority population of the country is underprivileged and faces financial issues to afford the costly liver transplant procedure. The other challenges include the emerging NASH and obesity epidemics. The prevalence of viral hepatitis has not decreased in the country despite advancements in antiviral therapies and vaccine availability against hepatitis B [7].

Other than the current challenges, future planning is of utmost importance. No local transplant-related statistics are available due to the lack of a liver transplant registry [6]. The long-term immunosuppression-induced adverse effects and the need for re-transplantation are still to face. The management of hepatocellular carcinoma (HCC) recurrence in patients transplanted for HCC will further add a burden on the health system which already has poor resources. In Pakistan, almost 5000 patients need liver transplantation every year and till now the maximum capacity of all the programs is to perform only 500 liver transplants. The major reason is that liver transplant in the private sector is way too costly and there are only a few government-funded programs in the country for the underprivileged [7]. The number and capacity of public sector programs need to be increased to overcome this huge burden.

The local transplant community needs to overcome the limitation of organ supply through various donor expansion approaches. This may include relaxation in the HOTA act for allowing non-blood related donation and swap transplantation, the provision of ABO-incompatible transplantation, and consideration of extended donor criteria. The recipient selection criteria also need to be reviewed for a good outcome and survival. This will minimize the undue or overuse of resources and the approach will be more focused on those individuals who are beneficial to society. The early detection of viral hepatitis by screening programs, and their prompt treatment with the latest antiviral drugs is likely to reduce the burden on the transplant waiting list. The provision of free immunosuppressive drugs to the recipients is vital for long-term outcomes.

To address all the above-mentioned challenges, the liver transplant registry is the need of the day, especially for the availability of accurate transplant-related statistics. Community education and legislation for the promotion of deceased organ donation is the ultimate roadmap to overcome the huge number of patients needing liver transplants per year in the country. Keeping in mind the socio-economic conditions of society, liver transplant in public sector hospitals is the only way out.

Although it may seem difficult to address all these challenges. Still, the transplant community and health authorities need to find an effective way to sort out all these challenges.

## Ethical approval

Not Applicable.

## Sources of funding

The author(s) received no financial support for the research, authorship, and/or publication of this article.

## Author contribution

Conceptualization and Methodology was done by AWD, Literature review was done by HBA, Manuscript was written by KU and SO, Review editing was done by AWD, Formatting and Referencing was done by KU and SO.

## Registration of research studies

Not Applicable.

## Guarantor

All authors take responsibility for the work, access to data and decision to publish.

## Consent

Not Applicable.

## Declaration of competing interest

The author(s) declared no potential conflicts of interest concerning the research, authorship, and/or publication of this article.
